# Variation in risk of opioid therapy and association with mortality following hip or knee arthroplasty: an analysis based on 14 different definitions

**DOI:** 10.2340/17453674.2025.44572

**Published:** 2025-09-02

**Authors:** Eskild Bendix KRISTIANSEN, Alma B PEDERSEN

**Affiliations:** 1 Department of Clinical Epidemiology, Aarhus University Hospital, Aarhus; 2 Department of Clinical Medicine, Aarhus University, Aarhus, Denmark

## Abstract

**Background and purpose:**

Long-term opioid therapy (LTOT) has frequently been reported in patients undergoing total hip or knee arthroplasty (THA or KA). However, there is no clear recommendation on the definition. We aimed to evaluate the sensitivity of the estimated risk of LTOT and association with mortality after THA and KA to the selection among 14 different candidate LTOT definitions.

**Methods:**

Using data from the nationwide Danish registries, we included patients with osteoarthritis undergoing primary THA during 2016–2019 (n = 28,957) or KA during 2014–2020 (n = 51,239). We obtained individual-level information on opioid prescriptions from any pharmacy 1 year before and 1 year after surgery. 14 common LTOT definitions were selected from the literature. The primary outcome was the variation in the 1-year crude risk of LTOT corresponding to variation in LTOT definition. Analysis was done overall and stratified by sex, age, prior opioid use, and year of surgery. The secondary outcome was the 4-year mortality among patients meeting each LTOT definition.

**Results:**

The 1-year risk of LTOT varied from 1.2% (95% confidence interval [CI] 1.1–1.3) to 20.1% (CI 19.6–20.5) for THA and 0.2% (CI 0.1–0.2) to 29.6% (CI 29.2–30.0) for KA patients depending on definition. For THA or KA, women had a higher risk of LTOT than men for all definitions, thus, LTOT varies from 0.2% (CI 0.1–0.2) to 32.9% (CI 32.3–33.4) for women and from 0.1% (CI 0.1–0.2) to 24.9% (24.4–25.5) for men. With increasing age risks of LTOT were steady or slightly decreasing. There was a decrease in the risk of LTOT from 2016 to 2019 for all definitions. 4-year mortality in patients meeting LTOT definitions varied from 9.8% (CI 8.9–10.7) to 16.3% (CI 13.2–20.1) for THA and 6.9% (CI 6.4–7.4) to 10.5% (CI 8.5–12.9) for KA patients.

**Conclusion:**

The estimation of the risk of LTOT after THA or KA and association with mortality is strongly dependent on the definition of LTOT used by researchers. This highlights the limitation on the comparability of opioid studies assessing risk and prognosis in these patients.

Patients receiving total hip arthroplasty (THA) and knee arthroplasty (KA) are routinely treated with opioids for acute pain in the week following surgery [1-4]. Proper pain management enhances rehabilitation and physical activity. However, some patients may be long-term opioid therapy (LTOT) users due to prolonged pain postoperatively [1,5]. This is a problem, because opioids have several adverse effects causing a substantial burden for patients, healthcare resources, and costs [6,7].

There is therefore great interest in studying the risk of LTOT in various populations, as well as the factors predicting which patients are at elevated risk, but a core issue that has not yet found wide agreement is the definition of LTOT.

Many candidate LTOT definitions are proposed in published literature [8,9], making the comparison between studies challenging. Further, it is unclear whether there are biases tied into the choice of definition itself and what characterizes patients included in specific LTOT, modifying the risk factors for and prognosis after use of various LTOT definitions.

Therefore, we primarily aimed to describe how the estimated risk of LTOT varies when employing 14 different LTOT definitions, and whether the variation in risk is more pronounced for categories of sex, age, prior opioid use, and year of surgery. The secondary aim was to describe the variation in mortality between patients meeting each individual LTOT definition.

## Methods

### Study design and setting

We conducted a population-based cohort study in Denmark, using data from the national medical registries [10]. Denmark provides tax-supported healthcare to all residents. Registries are linked through the unique civil registration number that is generated from the Civil Registration System and goes through all registries [11].

The study is reported with STROBE guidelines.

### Study population

In the risk of LTOT analysis, the study population consisted of patients with osteoarthritis receiving a primary THA or KA in the periods 2016–2019 (hips) and 2014–2020 (knees) due to data availability, counting the first procedure on either side individually. Patients were identified from the Danish Hip Arthroplasty Registry [12] and Danish Knee Arthroplasty Registry [13], which contains records of THA or KA (including both total and partial procedures) conducted in Denmark at public or private hospitals. Primary THA is a surgical procedure involving removal of diseased femoral head and acetabulum and replacing both with artificial implants, while primary KA involves surgical replacement of damaged both femoral and tibial components or 1 of them with artificial implants. The completeness of patient registration is more than 95% [14-16].

In the mortality analysis, the study population comprised 14 different subgroups obtained by restricting the main study population to those meeting each of the respective 14 LTOT definitions by the end of the first year of follow-up. Patients who died before this date or did not meet any LTOT definition were not included in the mortality analysis. Patients could be placed in multiple groups, if they met multiple LTOT definitions.

### Outcome

*Primary outcome—LTOT:* Information on dispensation of 13 common types of opioids was collected from the Danish National Prescription Registry [17], according to the Anatomical Classification System (ATC) codes, package size and doses, and dispensation dates (Supplementary Table). The Prescription registry contains information on all prescriptions dispensed by Danish residents from community pharmacies since 1995. The Prescription registry does not include information on hospital prescription dispensing.

We selected 14 different candidate definitions of LTOT from published literature [8,9]. Definitions were selected based on their frequency of use in the literature, measurability in registry data, categorical differences in opioid measurement methods, and clinical relevance to encompass a broad spectrum of available definitions. The LTOT definitions can be roughly categorized into 5 categories based on (i) number of prescriptions, (ii) number of days’ supply, (iii) oral morphine equivalent (OME) units, (iv) continuous episodes, or (v) some combination of i–iv. The OME values were calculated according to a set of conversion factors provided in Supplementary Table as reported by Nielsen et al., 2016 [18].

Opioids collected at the pharmacy within the first 7 days of THA or KA did not count toward any LTOT definition, due to the variance in each patient’s need for acute short-term pain management and local clinical guidelines.

*Secondary outcome—mortality:* Date of death due to any cause was obtained from the Civil Registration System.

### Covariates

Patient sex and age at the time of surgery were obtained from the Civil Registration System.

From the Danish Hip and Knee Arthroplasty Registries, we collected information on calendar year of surgery and body mass index (BMI).

From the Danish National Prescription Registry, we obtained information on opioid use prior to THA or KA, defined as ≥2 opioid prescriptions during the last 6 months leading up to surgery.

Somatic comorbidities 10 years before THA or KA were obtained from the Danish National Patient Registry. According to the International Classification of Diseases, 10th Revision (ICD-10) codes, we calculated a Charlson Comorbidity Index (CCI) score for each patient [19]. The comorbidity level was classified into 3 categories: low (CCI score 0), moderate (CCI score 1–2), or high (CCI score ≥3).

### Statistics

In the risk of LTOT analysis, we presented the patients’ characteristics as counts and percentages for categorical data and as median with interquartile range (IQR) for continuous data for the THA and KA study population separately. We calculated the overall 1-year risks of LTOT after THA or KA, according to each LTOT definition separately. The follow-up started 8 days after THA or KA surgery and ended 1 year after THA or KA surgery. The distribution of specific opioid types dispensed to the population during follow-up was reported in terms of the total sums of prescriptions. The risks of LTOT were stratified by sex, age, prior opioid use, and year of surgery. We estimated 95% confidence intervals (CI) for all LTOT risks using the Aalen–Johanssen estimator.

In the mortality analysis, we presented patients’ characteristics in each of the 14 LTOT definition subgroups. For each LTOT subgroup, we calculated crude 4-year mortality risk with 95% CI, starting follow-up at the end of the first year, to explore which definitions led to a closer association with mortality. Mortality analysis was done overall, and among male, female, and patients with low CCI, separately.

Study design may be seen in Supplementary Figure 1.

The analyses were performed using SAS version 9.4 (SAS Institute, Cary, NC, USA).

### Ethics, data sharing plan, funding, and disclosures

The study was reported to the Danish Data Protection Agency through registration at Aarhus University (record number: AU-2016-051-000001, sequential number 880). Ethical approval is not required in Denmark for studies based exclusively on routinely collected registry data. All data generated or analyzed during this study are included in this published article. According to the Danish legislation, datasets generated and/or analyzed in the current study are not publicly available. ABP was supported by, and employed at, the Department of Clinical Epidemiology at Aarhus University, and Aarhus University Hospital. The Department of Clinical Epidemiology, Aarhus University, and Aarhus University Hospital receive funding from various companies in the form of research grants to (and administered by) Aarhus University. None of these grants are related to the present study. The authors declare no conflicts of interest. Complete disclosure of interest forms according to ICMJE are available on the article page, doi: 10.2340/17453674.2025.44572

## Results

### Risk of LTOT analysis

**Figure 1:**
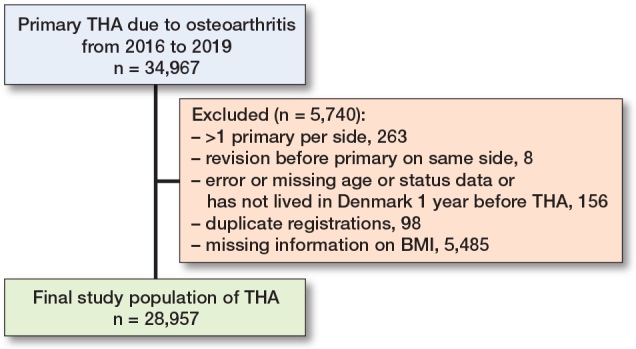
Flow diagram for THA (total hip arthroplasty) population.

**Figure 2:**
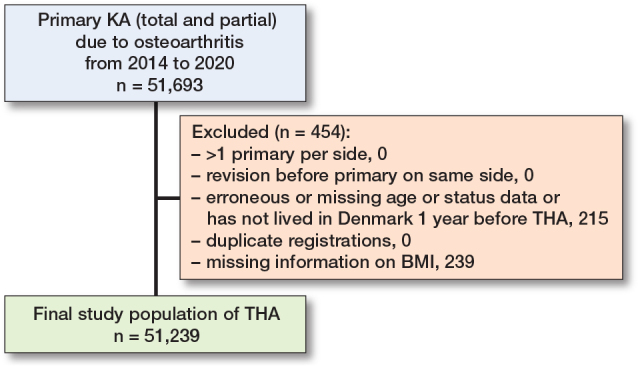
Flow diagram for KA (total and partial knee arthroplasty) population.

The study population included 28,957 THAs (26,644 patients) and 51,239 KAs (43,933 patients) (Figures 1 and 2). Most patients were female, and the average age was 70 years. BMI was slightly higher among KA than THA patients. Approximately 20% of both THA and KA patients were preoperative opioid users. 16.8% of THA and 17.2% of KA patients had a CCI score of 1 or more (Table 1). Patients with the contralateral side operated on had a slightly higher proportion of prior opioid users compared with patients who underwent a first procedure.

**Table 1:** Patients’ characteristics of the total hip arthroplasty (THA) and knee arthroplasty (KA) population used in the risk of long-term opioid therapy analysis. Values are count (%) or median (IQR)

	THA	KA
Number of surgeries	28,957	51,239
Female sex	16,307 (56)	30,207 (59)
Age at the time of surgery	71.1 (63.7–76.9)	70.0 (62.9–75.7)
Prior opioid use	6,263 (22)	10,002 (20)
BMI at the time of surgery	26.9 (24.2–30.3)	28.9 (25.8–32.8)
Charlson Comorbidity Index		
0	21,181 (73)	37,298 (73)
1	3,484 (12)	6,655 (13)
≥2	4,292 (15)	7,286 (14)

BMI: body mass index. IQR: interquartile range.

The 1-year risk of LTOT varied from 1.2% (CI 1.1–1.3) when defining LTOT as “180 days’ continuous use with < 7-day gap” to 20.1% (CI 19.6–20.5) when defining LTOT as “at least one prescription in Q2 or later” in the THA population. In the KA population, 1-year risk of LTOT varied from 0.2% (CI 0.1–0.2) when defining LTOT as “365 days of supply or > 18,000 OME with fill in every quarter” to 29.6% (CI 29.2–30.0) when defining LTOT as “3 or more prescriptions” (Table 2).

**Table 2:** Crude 1-year risk of long-term opioid therapy (LTOT) after total hip arthroplasty (THA) and knee arthroplasty (KA) with 95% confidence intervals (CI).

LTOT definitions	LTOT risk, n (%) [CI]	
	THA, n = 28,957	KA, n = 51,239
1) At least 1 prescription in Q2 or later	5,806 (20.1) [19.6–20.5]	14,768 (28.8) [28.4–29.2]
2) 3 or more prescriptions	4,541 (15.7) [15.3–16.1]	15,172 (29.6) [29.2–30.0]
3) >1,800 OME, with at least one prescription in Q2 or later	2,848 (9.8) [9.5–10.2]	8,666 (16.9) [16.6–17.2]
4) 6 or more prescriptions	2,406 (8.3) [8.0–8.6]	7,845 (15.3) [15.0–15.6]
5) 90 days of supply in total	1,532 (5.3) [5.0–5.6]	4,344 (8.5) [8.2–8.7]
6) 90 days continuous use with <30-day gap	1,562 (5.4) [5.1–5.7]	4,689 (9.2) [8.9–9.4]
7) At least 1 prescription in each quarter	1,616 (5.6) [5.3–5.9]	4,465 (8.7) [8.5–9.0]
8) 180 days of supply or >4,500 OME spread over at least 3 different quarters	1,561 (5.4) [5.1–5.7)	4,493 (8.8) [8.5–9.0]
9) >120 days’ supply or >10 fills in a 90-day window	1,295 (4.5) [4.2–4.7]	3,731 (7.3, ) [7.1–7.5]
10) 180 days continuous use with <30-day gap	873 (3.0) [2.8–3.2]	2,455 (4.8) [4.6–5.0]
11) 180 days of supply in total	770 (2.7) [2.5–2.9]	2,175 (4.2) [4.1–4.4]
12) 90 days continuous use with <7-day gap	609 (2.1) [1.9–2.3]	1,863 (3.6) [3.5–3–8]
13) 180 days continuous use with <7-day gap	351 (1.2) [1.1–1.3]	1,093 (2.1) [2.0–2.3]
14) 365 days of supply or >18,000 OME with fill in every quarter	404 (1.4) [1.3–1.5]	85 (0.2) [0.1–0.2]

CI: 95% confidence interval; Q1–4: 1st through 4th quarter after procedure; OME: oral morphine equivalents.

The distribution of most common prescribed opioids in the THA population was morphine 19%, oxycodone 43%, tramadol 29%, and fentanyl 2%, while distribution in the KA population was morphine 19%, oxycodone 48%, tramadol 27%, and fentanyl 1%.

**Figure 3:**
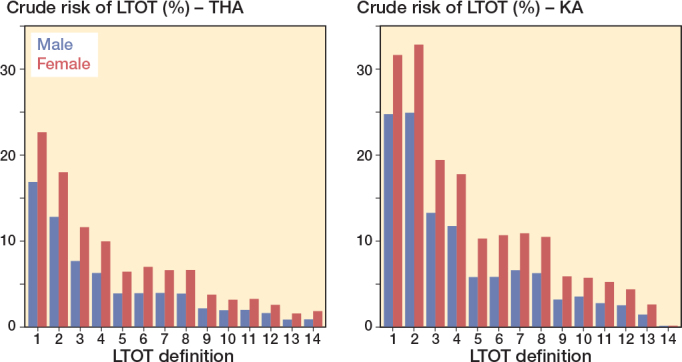
1-year crude risks of long-term opioid therapy (LTOT) after total hip arthroplasty (THA) and knee arthroplasty (KA) by sex.

Female patients had a higher risk of LTOT than male patients no matter how LTOT was defined and in both the THA and the KA population (Figure 3). Thus, in female patients, risks of LTOT varied from 1.5% (CI 1.3–1.7) to 22.6% (CI 22.0–23.2) after THA and from 0.2% (CI 0.1–0.2) to 32.9% (CI 32.3–33.4) after KA depending on the LTOT definition (Figure 3). In male patients, risks of LTOT varied from 0.8% (CI 0.7–1.0) to 16.8% (CI 16.2–17.5) after THA and from 0.1% (CI 0.1–0.2) to 24.9% (CI 24.4–25.5) after KA (Figure 3). The highest 1-year risks of LTOT were estimated when LTOT was defined as “at least one prescription in Q2 or later” or as “3 or more prescriptions” in both female and male patients.

**Figure 4:**
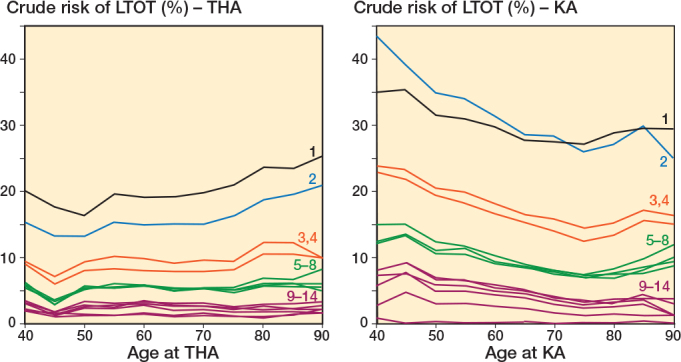
1-year risks of LTOT after THA and KA by age. For abbreviations, see Figure 3.

In the THA population, increasing age was associated with a constant or slightly decreasing risk of LTOT for 12 definitions, whereas increasing age was associated with increasing risk of LTOT for 2 LTOT definitions (LTOT defined as “at least one prescription in Q2 or later” or as “3 or more prescriptions”) (Figure 4). In the KA population, increasing age was associated with steady or decreasing risk of LTOT for all definitions, showcasing an example of definition-specific bias (Figure 4).

**Figure 5:**
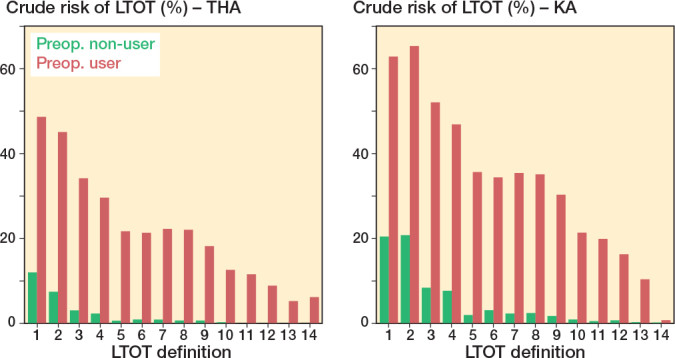
1-year risks of LTOT after THA and KA by preoperative opioid use. For abbreviations, see Figure 3

Preoperative opioid users had a higher risk of LTOT than non-users irrespective of LTOT definition in both the THA and KA population. Among both preoperative opioid users and non-users, the highest risk of LTOT was seen when LTOT was defined as “at least one prescription in Q2 or later” or “3 or more prescriptions”, being 45.1% (CI 43.9–46.3) and 48.7% (CI 47.4–49.9) in preoperative users and 7.6% (CI 7.2–7.9) and 12.2% (CI 11.7–12.6) in preoperative non-users in the THA cohort, and 63.2% (CI 62.2–64.1) and 65.7% (CI 64.7–66.6) in preoperative users and 20.5% (CI 20.1–20.9) and 20.9% (CI 20.5–21.2) in preoperative non-users in the KA cohort. For other LTOT definitions, risks of LTOT varied from 0.7% (CI 0.5–0.9) to 52.3% (CI 51.3–53.3) among preoperative opioid users, while among opioid non-users risks of LTOT varied from 0.0% (CI 0.0–0.1) to 8.3% (CI 8.1–8.6) (Figure 5).

**Figure 6:**
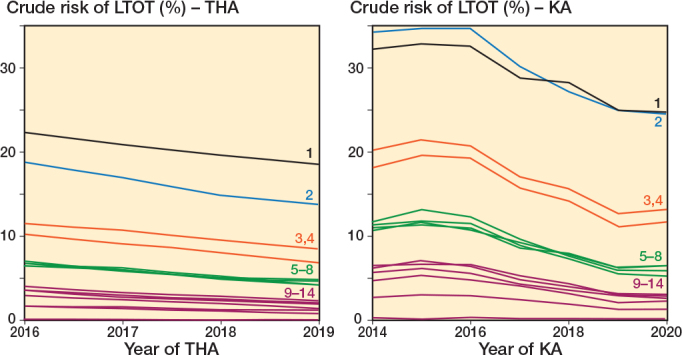
1-year risks of LTOT after THA and KA by year of surgery. For abbreviations, see Figure 3.

During the period 2016–2019 for THA and 2014–2020 for KA patients, we observed a decreasing risk of LTOT for almost all definitions and in both populations (Figure 6).

### Mortality analysis

The study populations included patients meeting each of the 14 LTOT definitions. Patients’ characteristics by 14 LTOT definition groups are presented in Table 3.

**Table 3:** Patients’ characteristics of 14 long-term opioid therapy (LTOT) definition groups used in the mortality analysis separate for total hip arthroplasty and knee arthroplasty patients. Values are count (%) or median (IQR)

LTOT definition	Median age (IQR)	Female sex	Prior opioid use	Median BMI (IQR)	CCI 0	CCI 1	CCI ≥2
Total hip arthroplasty patients							
1	71.9 (64.5–77.7)	3,683 (63)	3,051 (53)	28 (25–31)	3,756 (65)	892 (15)	1,158 (20)
2	71.9 (64.5–78.0)	2,927 (65)	2,825 (62)	28 (25–32)	2,842 (63)	751 (17)	948 (21)
3	71.7 (63.9–77.8)	1,884 (66)	2,136 (75)	28 (25–32)	1,695 (60)	499 (18)	654 (23)
4	71.9 (64.1–77.9)	1,618 (67)	1,859 (77)	28 (25–32)	1,426 (60)	432 (18)	548 (23)
5	71.0 (63.6–76.8)	1,045 (68)	1,366 (89)	28 (25–32)	913 (60)	263 (17)	356 (23)
6	71.2 (63.8–77.0)	1,068 (68)	1,339 (86)	28 (25–33)	931 (60)	260 (17)	371 (24)
7	71.8 (63.9–78.0)	1,130 (70)	1,400 (87)	28 (25–32)	961 (60)	283 (18)	372 (23)
8	71.4 (63.5–77.2)	1,073 (69)	1,390 (89)	28 (25–32)	931 (60)	267 (17)	363 (23)
9	71.1 (63.7–76.5)	885 (68)	1,146 (89)	28 (25–32)	767 (59)	226 (18)	302 (23)
10	70.8 (63.4–76.4)	605 (69)	796 (91)	28 (25–33)	517 (59)	144 (17)	212 (24)
11	70.4 (63.1–75.7)	524 (68)	734 (95)	28 (25–32)	452 (59)	125 (16)	193 (25)
12	70.4 (62.5–75.7)	410 (67)	569 (93)	28 (25–33)	348 (57)	106 (17)	155 (26)
13	71.2 (62.5–76.4)	247 (70)	333 (95)	28 (24–33)	198 (56)	62 (18)	91 (26)
14	70.9 (63.1–75.8)	298 (74)	387 (96)	28 (25–33)	224 (55)	70 (17)	110 (27)
Knee arthroplasty patients							
1	69.6 (62.1–75.6)	9,558 (65)	6,319 (43)	30 (26–34)	10,002 (68)	2,344 (16)	2,422 (16)
2	69.1 (61.6–75.0)	9,926 (65)	6,570 (43)	30 (26–34)	10,347 (68)	2,384 (16)	2,441 (16)
3	68.8 (61.4–74.9)	5,869 (68)	5,232 (60)	30 (26–34)	5,652 (65)	1,471 (17)	1,543 (18)
4	68.6 (61.1–74.7)	5,374 (69)	4,707 (60)	30 (26–34)	5,135 (66)	1,351 (17)	1,359 (17)
5	68.2 (60.9–74.5)	3,116 (72)	3,576 (82)	30 (26–35)	2,725 (63)	802 (19)	817 (19)
6	68.0 (60.5–74.3)	3,294 (70)	3,448 (74)	30 (26–35)	2,999 (64)	838 (18)	852 (18)
7	68.7 (61.2–75.1)	3,230 (72)	3,553 (80)	30 (26–34)	2,787 (62)	822 (18)	856 (19)
8	68.3 (60.8–74.6)	3,174 (71)	3,528 (79)	30 (26–34)	2,807 (63)	840 (19)	846 (19)
9	67.9 (60.5–74.1)	2,653 (71)	3,040 (82)	30 (27–34)	2,359 (63)	678 (18)	694 (19)
10	67.2 (59.8–73.9)	1,781 (73)	2,141 (87)	30 (27–35)	1,510 (62)	462 (19)	483 (20)
11	67.1 (60.0–73.5)	1,580 (73)	1,989 (91)	31 (27–35)	1,340 (62)	417 (19)	418 (19)
12	66.8 (59.5–73.3)	1,326 (71)	1,625 (87)	31 (27–35)	1,152 (62)	358 (19)	353 (19)
13	66.3 (59.4–72.7)	794 (73)	1,030 (94)	31 (27–35)	667 (61)	215 (20)	211 (19)
14	67.5 (63.3–74.4)	54 (64)	68 (80)	30 (27–34)	53 (62)	15 (18)	17 (20)

BMI: body mass index, CCI: Charlson Comorbidity Index; IQR: interquartile range

In THA patients, the prevalence of females varied from 63.4% to 73.8%, median age varied from 71–72 years, prevalence of CCI score 0 varied from 55.4% to 59.6%, and prevalence of prior opioid users varied from 52.5% to 95.5% by 14 LTOT definition groups. Similar variation was seen in the KA population (Table 3).

**Figure 7:**
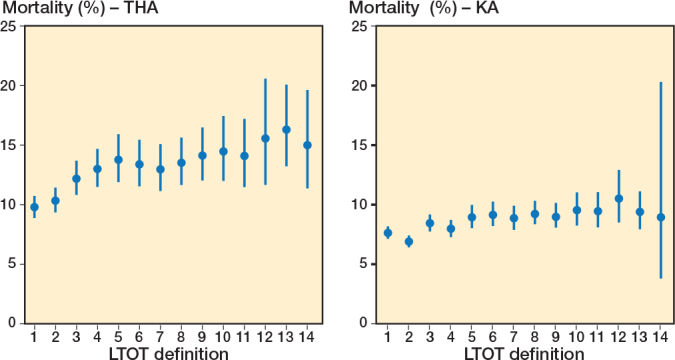
4-year mortality in patients fulfilling LTOT definitions in THA and KA patients. For abbreviations, see Figure 3.

The 4-year mortality among patients meeting different LTOT definitions varied from 9.8% (CI 8.9–10.7) when defining LTOT as “at least one prescription in Q2 or later” to 16.3% (CI 13.2–20.1 ) when defining LTOT as “90 days of continuous use with < 7 day gap” in the THA population, and from 6.9% (CI 6.4–7.4) when defining LTOT as “3 or more prescriptions” to 10.5% (CI 8.5–12.9) when defining LTOT as “180 days’ continuous use with < 7 day gap” in the KA population (Figure 7). Patients meeting LTOT definitions leading to lower mortality comprise slightly more patients who are less comorbid and have low prior opioid use compared with patients meeting LTOT definitions leading to higher mortality (see Table 3). There was no clear correlation between distribution of first and second procedure and LTOT definitions leading to lower or higher mortality. The 4-year mortality among male and female patients separately meeting different LTOT definitions showed a similar pattern to that seen in overall mortality analysis. Likewise, there was no specific LTOT definition among patients with low CCI that was exhibiting different mortality risk than observed in overall mortality analysis (Supplementary Figures 2 and 3). However, all mortality estimates were slightly lower in patients with low CCI irrespective of LTOT definition than in overall mortality analysis.

## Discussion

We aimed to evaluate the sensitivity of the estimated risk of LTOT and association with mortality after THA and KA to the selection among 14 different candidate LTOT definitions.

We found significant variability in the estimation of risk of LTOT following THA and KA based on different LTOT definitions. The 1-year risk of LTOT ranged widely from 1.2% to 20.1% for THA and 0.2% to 29.6% for KA. The crude mortality varied from 6.5% to 14.8% by LTOT definition in THA or KA patients. This variability underscores the lack of a standardized definition for LTOT, which complicates the comparison of results across different studies.

We observed that women consistently exhibited a higher risk of LTOT compared with men across all definitions, which is in accordance with previous findings [3,20-22]. This sex disparity may be attributed to differences in pain perception, sociocultural and psychological factors [23,24], and warrants further investigation. Sex-specific approaches to pain management and opioid prescription may be necessary. Clinicians should be aware of these differences and consider alternative pain management strategies to reduce the reliance on opioids, particularly in female patients.

Our findings also indicate a decreasing trend in LTOT risk from 2016 to 2019 for all definitions, which has been seen in other studies [1,22]. This decline could reflect changes in clinical practice, such as increased awareness of opioid-related side effects, implementation of opioid-sparing protocols, or enhanced postoperative pain management strategies [25]. This is encouraging and suggests that efforts to reduce opioid use in postoperative settings are having a positive impact. Continued emphasis on opioid-sparing protocols, patient education, and multimodal pain management strategies will be crucial in sustaining this trend.

Our findings on mortality suggest that LTOT, irrespective of definition, is associated with a substantial mortality risk, which is in agreement with current knowledge [7] emphasizing the need for careful patient monitoring and management. In addition, some LTOT definitions are prone to capture more sick patients measured with prior opioid use and a comorbidity burden known to be associated with increased mortality [26]. Our findings of consistently higher mortality after THA than KA could also be explained by the fact that the prevalence of prior opioid use and comorbidity burden was higher for THA patients meeting almost all of the 14 LTOT definitions than for KA patients.

### Limitations

For this study, we were able to include every THA and KA in Denmark in their respective periods, leaving little reason to worry about selection bias.

However, as the Prescription Registry does not contain information on the indication for prescription, we are not sure if the patient is receiving opioids for postoperative pain or for pain due to some other indication. We have attempted to compensate for this by stratifying the cohorts on preoperative opioid use. Also, while we know exactly what patients have collected which opioids from the pharmacy, the compliance with treatment is unknown.

We have not conducted mediation analyses to account for subsequent surgeries or reoperations or other events during follow-up that might affect opioid use. As THA and KA are major surgeries, new planned surgeries during the 1st year of follow-up should be quite rare, in order not to interfere with the rehabilitation process. This is, however, not the case for acute surgeries. It is possible that painful events within 1 year of THA or KA could have impacted the observed risks of LTOT and that the size of impact varies by LTOT definition. However, variations in LTOT risks in our stratified analyses were similar to variation observed in the overall THA or KA population suggesting that painful events were of minor importance to our study results.

### Conclusion

Our study demonstrates that the risk of LTOT and association with mortality after THA or KA is highly dependent on the LTOT definition used. The risk of LTOT varies from 0.2% to 29.6%. Variation in mortality from 6.9% to 16.3% suggests that definitions capture patients with different health statuses.

*In perspective,* the observed variation in LTOT risk has important implications across clinical and policy domains. Divergent definitions can lead to patient misunderstandings, inconsistent prescribing practices, and fragmented policymaking. Standardizing LTOT definitions and adoption of tailored clinical approaches can enhance communication, research comparability, and patient outcomes, and reduce the burden of LTOT. We encourage researchers to clearly report which LTOT definition they use and provide a rationale for their choice. Additionally, we suggest applying sensitivity analyses, and changing the LTOT definition to assess the impact of the LTOT definition on study results.

### Supplementary data

Supplementary Table and Supplementary Figures 1–3 are available on the article page, doi: 10.2340/17453674.2025.44572

## Supplementary Material



## Notes

EK and ABP contributed substantially to conception and study design, data acquisition, analyses plan, interpretation of data, drafting and revising the paper, finalizing the paper, final approval of the paper, and are accountable for all aspects of the work.

Handling co-editors: Marianne Westberg and Robin Christensen

*Acta* thanks Torbjørn Berge Kristensen and Carl-Olav Stiller for help with peer review of this manuscript.
