# Supplementary material for: Variation in risk of opioid therapy and association with mortality following hip or knee arthroplasty: an analysis based on 14 different definitions
Source: Acta Orthop. 2025 Sep 2;96:664–70. doi: 10.2340/17453674.2025.44572 (PMC12404099; doi:10.2340/17453674.2025.44572)

SUPPLEMENTARY DATA

Supplementary Table 1. The Anatomical Classification System codes (ATC) used to identify opioids and oral morphine equivalent (OME) conversion factors used [17]

Drug	ATC	Administration method	Conversion factor
**Morphine**	**N02AA01**	Oral	1.0
	**N02AA01**	Parenteral	3.0
**Hydromorphone**	**N02AA03**	Oral	4.0
**Oxycodone**	**N02AA05**	Oral	1.5
		Parenteral	3.0
**Tramadol**	**N02AX02**	Oral	0.2
**Tapentadol**	**N02AX06**	Oral	0.4
**Codeine**	**R05DA04**	Oral	0.15
**Pethidine**	**N02AB02**	Oral	0.4
**Buprenorphine**	**N02AE01**	Parenteral	75
	**N02AE01**	Sublingual	10
	**N02AE01**	Transdermal	75
**Fentanyl**	**N02AB03**	Sublingual	130
	**N02AB03**	Transdermal	100

Supplementary Figure 1. Study design. THA: total hip arthroplasty, KA: knee arthroplasty, LTOT: long-term opioid therapy.

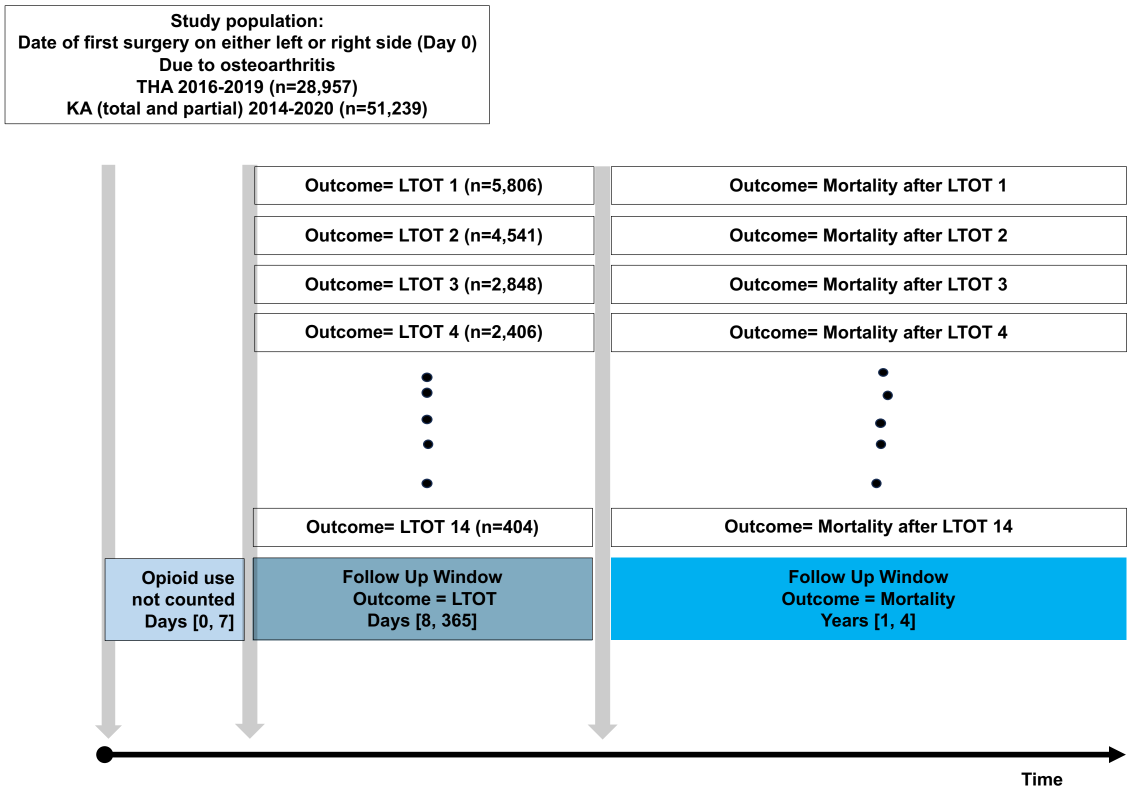

Supplementary Figure 2. 4-year mortality in patients fulfilling LTOT definitions in THA and KA patients separately for male and female patients. For abbreviations, see Supplementary Figure 1.

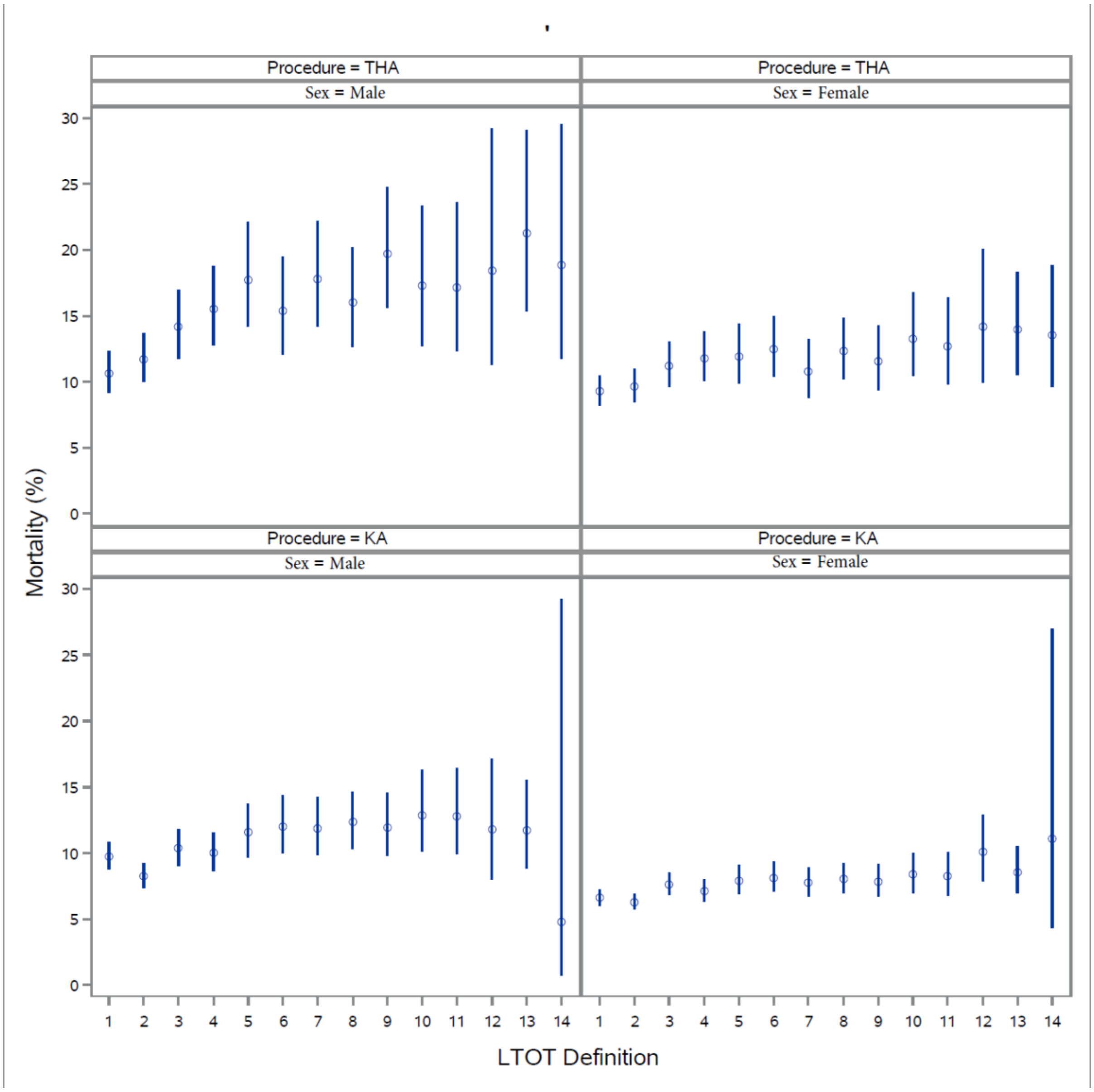

Supplementary Figure 3. 4-year mortality in patients fulfilling LTOT definitions in THA and KA patients separately for patients with low Charlson comorbidity index (CCI). For abbreviations, see Supplementary Figure 1.

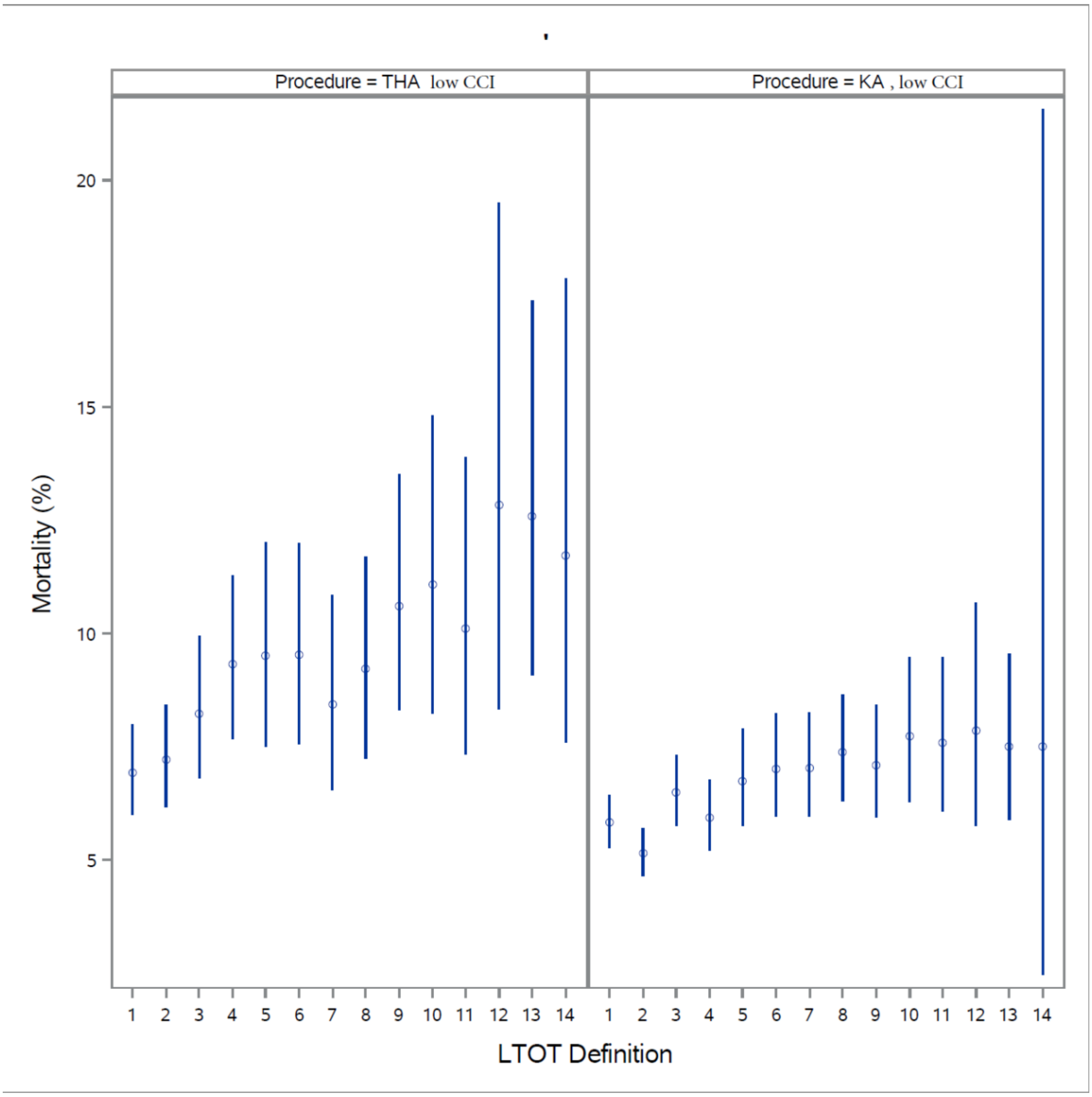

Supplement: Supplementary file 1 [file ActaO-96-44572-s1.pdf]
